# Measurement of Particle Size of Loose Accumulation Based on Alpha Shapes (AS) and Hill Climbing-Region Growing (HC-RG) Algorithms

**DOI:** 10.3390/s20030883

**Published:** 2020-02-07

**Authors:** Yunfeng Ge, Zishan Lin, Huiming Tang, Peng Zhong, Bei Cao

**Affiliations:** 1 Faculty of Engineering, China University of Geosciences, Wuhan 430074, China; geyunfeng@cug.edu.cn (Y.G.); cbei@cug.edu.cn (B.C.); 2 Three Gorges Research Center for Geo-Hazard, Ministry of Education, China University of Geosciences, Wuhan 430074, China; 3 School of Earth Sciences and Engineering, Nanjing University, Nanjing 210046, China; zhongpeng@smail.nju.edu.cn

**Keywords:** particle size measurement, loose accumulation, alpha shapes (AS) algorithm, hill climbing-region growing (HC-RG) algorithm, laser scanning

## Abstract

The loose accumulation CAUSED by landslide, collapse, debris flow, and mine blasting, exerts considerable negative influence to human activities. Besides, it can easily trigger secondary disaster under inner and outer geological conditions. Extraction and measurement of the particle of loose accumulation is of importance for prediction of slope stability and mine blasting. In this paper, the 3D laser scanning is utilized to collect the point clouds of granular materials in physical model (three types of materials) and landslide accumulation in field, respectively. Then, the alpha shapes (AS) and hill climbing-region growing (HC-RG) algorithms are introduced for identifying particles and finding their dimensions (e.g., particle number and radii). Comparison between the recognition results and reality shows that both algorithms can provide a good performance in laboratory physical model, and acceptable results can be obtained when applying two algorithm to field survey. AS algorithm needs less time to process data than HC-GR algorithm; however, the recognition from HC-RG algorithm is more accurate than that by AS algorithm.

## 1. Introduction

Loose accumulation, caused by landslide, collapse, debris flow, and mine blasting, poses a huge threat to human life and property. It tends to trigger more concatenated hazards and risks under the conditions of rainfalls, water level fluctuation of reservoirs, and earthquakes, which in turn trigger further hazards to form an interconnected hazard network [1,2,3,4,5]. Loose accumulation can be defined as an assembly of granular materials without too much compaction; measuring the particle shape and distribution is a fundamental task in various fields. In geohazards, the deposit (loose accumulation) characteristics provide important and useful implications necessary to understand the landslide failure model and evolution mechanism, and are considered necessary parameters to describe the impact region of hazards [6,7,8]. In mining industry, rock fragmentation generated by blasting is also a kind of loose accumulation, and the particle size distribution has a direct impact on the downstream process of mining, including transportation, crushing, grinding, and industrial costing control [9]. In civil engineering, the quality of concrete and compactness of filled earth highly depend on the particle size distribution of building materials used in the construction [10,11]. In sedimentology, the structure of gravel beach accumulation is regarded as a good indicator in the geological record, which records, in details, the information about the geological structure changing and paleoenvironmental evolution [12]. Therefore, accurately recognizing the particles from loose accumulation is an important and meaningful study topic [13,14]. To date, many powerful tools are available to study the particle size distribution both in microscopic and macroscopic scale, such as Scanning Electron Microscopy (SEM) [15] and image segmentation [16,17]. However, there are some limitations based on the 2D geometric information, leading to measurement error. In fact, in the loose accumulation of engineering construction and geological disasters, the particles may be a combination of giant, medium, and fine mass. Due to the complexity of particle shape, the traditional methods are not capable of good performance with high efficiency and resolution. Fortunately, with 3D laser scanning, a newly-developed and non-contact measurement, we can collect the point cloud of object surface in an accurate and fast way. Therefore, this technology can realize the acquisition and reconstruction of topography for complex and irregular geometries [18]. Besides, laser scanning is widely used in deformation monitoring for slope and construction, but it rarely appears in particle analysis field [19,20,21].

With the rapid development of artificial intelligence (AI), the focus on digital image processing for loose accumulation analysis has been evolving from basic applications to the development of intelligence algorithms to detect loose accumulation and extract parameters automatically or semi-automatically [22,23,24]. Currently, several researchers pay more attention to alpha shapes (AS) and region growing (RG) algorithms, which are regarded as effective tools to recognize boundary of desired objects [25,26,27]. The AS algorithm can reconstruct the non-convex hull of a point cloud depending on the single *α* value, and extract the geometric features more accurately [28]. The RG algorithm is an image segmentation method to separate the interested objects from the background by examining the difference between the seed and neighboring points based on similarity criterion [29]. However, it is difficult and time-consuming to specify the seed points, which are closely related to the calculation results, from the huge number of points when applying RG into particle recognition. To enhance the computational efficiency, the hill climbing (HC) algorithm is employed to find the highest point as the vertex of the local region points, and the hill increasing-region growing (HC-RG) algorithm is proposed to detect the particles from point cloud of deposit.

This article aims to develop an automatic procedure to detect particles in loose accumulation and measure their geometric parameters from point cloud acquired by 3D laser scanning. A laboratory physical model and field survey in a real landslide hazards were selected to test the applicability of AC and HC-RG algorithms presented in this study.

## 2. Methods

### 2.1. Data Collection

#### 2.1.1. Data Collection in Laboratory Physical Model

A physical model is a powerful tool to reproduce the process of landslide failure and reveal general regulation of real landslide hazards in spite of lacking in dynamic similarity [30]. Therefore, the physical model (flume testing) was used to simulate the landslide loose accumulation in the laboratory, and then a laser scanner was employed to collect the point cloud of accumulation.

**Figure 1:**
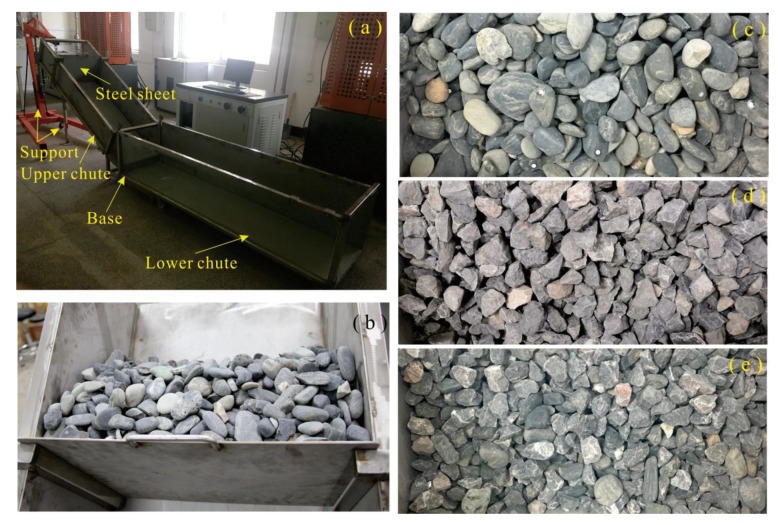
The experiment equipment and sliding materials used in physical model: (**a**) flume, (**b**) steel sheet, (**c**) pebbles, (**d**) gravels, and (**e**) mixtures.

The flume consists of an upper chute, a lower chute, a base, and a support (jack). One side of lower chute is made of stlinite for the observation purpose, and SUS304 stainless steel is chosen for other parts of flume. The upper chute is 1.5 m long, 0.5 m wide, and 0.3 m high, whereas the lower chute has dimensions of 2 m (Length) × 0.5 m (Width) × 0.4 m (Height). The inclination of upper chute can be changed by adjusting the hydraulic bottle jack. There is a steel sheet seated on the upper chute whose position can be altered along the chute to set different volume of sliding body of landslide. To test the reliability of proposed algorithms, three different types of materials were selected as sliding body, including pebble, gravel, and mixed material. The river pebbles, a natural material, feature smooth surface and good psephicity with relatively consistent shapes. The gravel, which is crushed and graded by screens, is characterized by irregular and angular geometry. The third material is created by mixing both of pebbles and gravels. The sliding materials size roughly between 5 mm and 40 mm, which were placed behind the steel sheet released through taking out the steel sheet (Figure 1).

**Figure 2:**
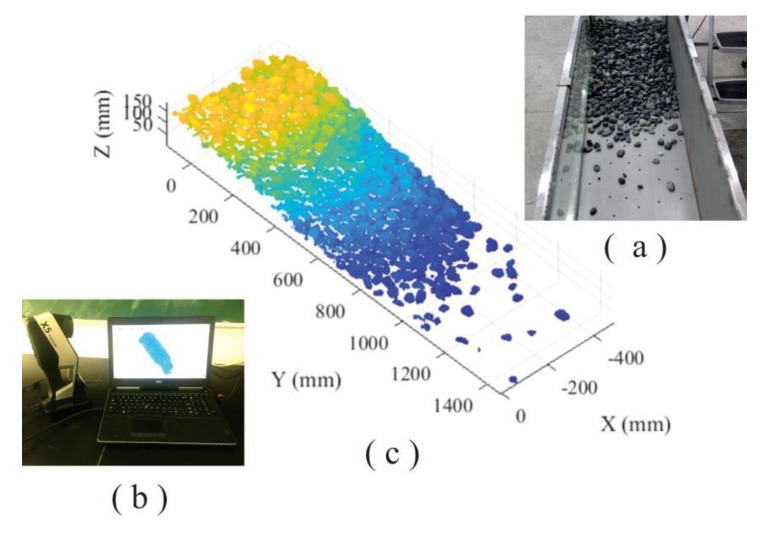
The collection of point cloud in laboratory physical model: (**a**) particle loose accumulation, (**b**) portable laser scanner, and (**c**) point cloud of accumulation.

In laboratory testing, the volume, height, and inclination angle of sliding materials was specified as 46,080 cm^3^, 110 cm, and 38°, respectively. A portable laser scanner—3D OKIO-X5 free laser scanner—was utilized to collect dense point clouds of deposit. According to the surrounding environment and color of scanned objects, the lightness level of light-emitting diode (LED) was specified as 300 and optical intensity was set as 500, respectively. In this manner, the point cloud has enough resolution (average point interval = 0.1 mm) to accurately reflect the detailed geometrical characteristics of loose accumulation surface (Figure 2).

#### 2.1.2. Study Area and Data Collection in Field Survey

**Figure 3:**
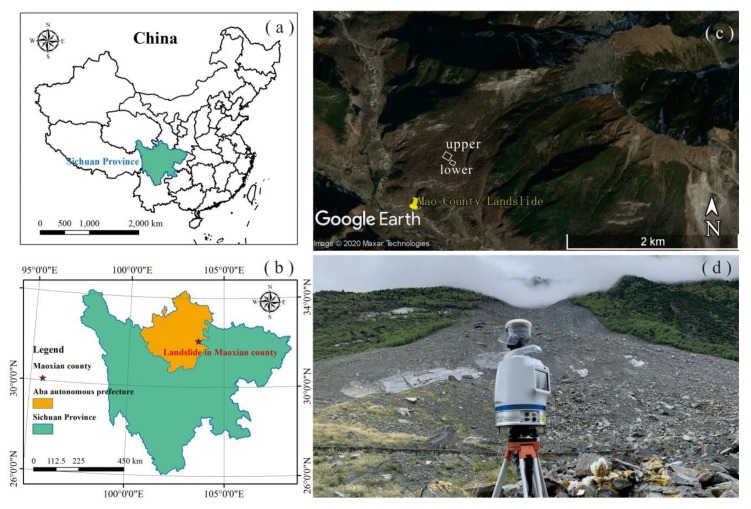
Location of Mao County landslide (**a**,**b**) and scanning regions in the accumulation (**c**). Field configuration of the Optech Polaris LR laser scanner used to collect the dense point cloud of landslide deposit (**d**).

The landslide, with coordinates of 103°39′ E and 32°04′ N, happened on 24 June 2017 in the back mountain of Xinmo Village, Diexi Town, Mao County, Sichuan Province, China. This place locates in the hinterland of Hengduan Mountains, and at the left bank of Songping valley, which is a branch of Minjiang River. The failure materials slid down from the top of mountain with with 3450 m average elevation and travelled a horizontal distance of about 2800 m within 100 s, causing 83 people dead. The rock type in this region is dominated by jointed quartzite, which tends to fracture and break due to the collision and friction in landslide failure processing [31,32]. Two locations of landslide accumulation—upper part and lower part—were selected for particle size analysis, and a terrestrial laser scanner (TLS), Optech Polaris LR, was employed to collect the dense point clouds (interval was specified as 0.05 m) of landslide accumulation in this two regions (Figure 3).

### 2.2. Alpha Shapes (AS) Algorithm

**Figure 4:**
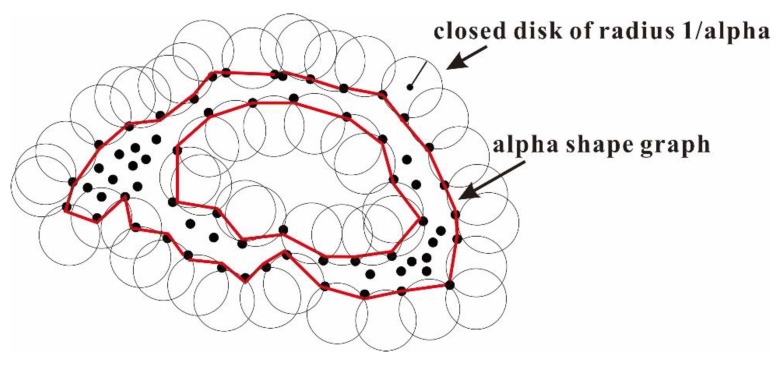
Schematic diagram of alpha shapes algorithm (modified after work in [34]).

There are many voids among the particles in the loose accumulation, and particles are not in a close physical contact with each other. The information about these voids inside of accumulation tends to be ignore using convex algorithms to determine the envelope of this type of point clouds. The alpha-shape algorithm, a well-known non-convex algorithm, is a powerful tool to accurately determine the alpha hull of a given set of points. The alpha hull can be considered as the boundary created by rolling a disk with a radius of 1/alpha over the points (Figure 4). The alpha value is a key parameter, and when it is specified as 0, the disk will have an infinite radius, and the alpha hull of the given points will equal to the convex hull. For a given alpha value, the alpha hull of point cloud associated with loose accumulation is reconstructed as follows. First, a vertex is created for each point in the given point cloud. Second, the edge between two vertices was generated when meeting two properties. Then the connected edges are defined as the boundary of point set [33,34].

### 2.3. Hill Climbing-Region Growing (HC-RG) Algorithm

Based on certain criterion (e.g., color, intensity, and point normal), the region growing algorithm can correctly segment regions that share same defined properties. One of disadvantages for region growing is lacking a method to determine the initial growing seed for landslide accumulation. This defect can be addressed by block contraction and local growth that consider the global view of the problem in HC-RG algorithm.

First, the neighboring point cloud is searched to form fitting the local neighborhood least square plane for each point according to the surface digitized data of particles [35]. The neighborhood point set is obtained by selecting m adjacent points for each point Pi in the point cloud.

The neighborhood point set was obtained by selecting m adjacent points for each point Pi in the point cloud. Each point cloud is represented as ${\left[X_{1},X_{2},X_{3}\right]}^{T}$, each point cloud representation is denoted as $P_{i}={\left[X_{1i},X_{2i},X_{3i}\right]}^{T}\left(i=1,2,\cdots ,m\right)$. Combining all the point cloud variables into a matrix:

(1) $$X={\left[\begin{array}{c} X_{11}\\ X_{21}\\ X_{31} \end{array}\;\begin{array}{c} X_{12}\\ X_{22}\\ X_{23} \end{array}\;\begin{array}{c} \cdots \\ \cdots \\ \cdots \end{array}\;\begin{array}{c} X_{1m}\\ X_{2m}\\ X_{3m} \end{array}\right]}_{3\times m}$$

Under normal circumstances, the mass center K point can be regarded as the center point of all points in a point set: $K=\frac{1}{n}\sum_{i=1}^{m}x_{i}$, at the same time $y_{i}=X_{i}-K$. The original point cloud matrix subtracts the mean value ${\left[X_{1K},X_{2K},X_{3K}\right]}^{T}$ in the corresponding direction, the mean value was calculated from local point cloud, and then a new point cloud variable matrix is reconstructed.

(2) $$Y={\left[\begin{array}{c} X_{11}\\ X_{21}\\ X_{31} \end{array}\;\begin{array}{c} X_{12}\\ X_{22}\\ X_{23} \end{array}\;\begin{array}{c} \cdots \\ \cdots \\ \cdots \end{array}\;\begin{array}{c} X_{1m}\\ X_{2m}\\ X_{3m} \end{array}\right]}_{3\times m}-\left[\begin{array}{c} X_{1K}\\ X_{2K}\\ X_{3K} \end{array}\right]={\left[\begin{array}{c} Y_{11}\\ Y_{21}\\ Y_{31} \end{array}\;\begin{array}{c} Y_{12}\\ Y_{22}\\ Y_{23} \end{array}\;\begin{array}{c} \cdots \\ \cdots \\ \cdots \end{array}\;\begin{array}{c} Y_{1m}\\ Y_{2m}\\ Y_{3m} \end{array}\right]}_{3\times m}$$

It is shown in Equation (3) that covariance matrix of the correlation between variables of point set in local region.

(3) $$S=\frac{1}{m}YY^{T}$$

The shortest distance from all point clouds to the fitted local region can be simplified to calculating the minimum eigenvalue of the covariance matrix.

Eigenvalues and eigenvectors:

(4) $$S_{n}=\lambda n=\left(\lambda E-S\right)n=0$$

Characteristic equation:

(5) det(λE−S)n=0

The corresponding eigenvector is solved, and the eigenvector of unitization corresponding to the minimum eigenvalue is selected, which is the point normal vector of the point.

**Figure 5:**
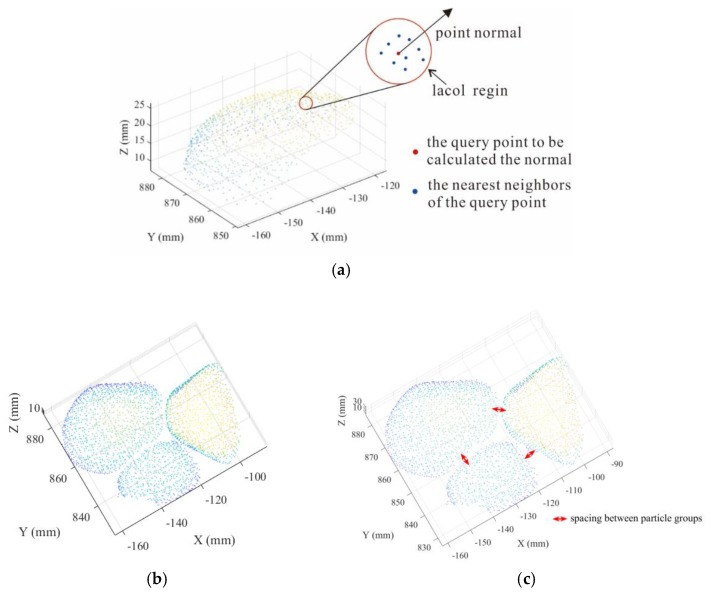
Schematic diagram of block separation; (**a**) definition of point normal, (**b**) original block point cloud, and (**c**) block point cloud of after separation.

Then, the normal vector of a point is calculated as the normal vector of this region, and the point is moved along the reverse direction of normal vector. After the movement for each point completed, the separation of point cloud data is realized (Figure 5). In the separation process, the quantity of neighboring point cloud influences the accuracy of point normal vector for questionable points, which means a small quantity cannot represent its point normal vector and large quantity will produce error for point normal vector. Moreover, the moving distance of the point influence the shapes and sizes of particles after contraction, which implies that a short distance will hinder accurate identification between blocks and long distance will change shapes and sizes of blocks. Therefore, we need to choose appropriate quantity of neighboring point cloud and moving distance. Besides, restitution in integrum is needed when calculating particle radius for accuracy.

**Figure 6:**
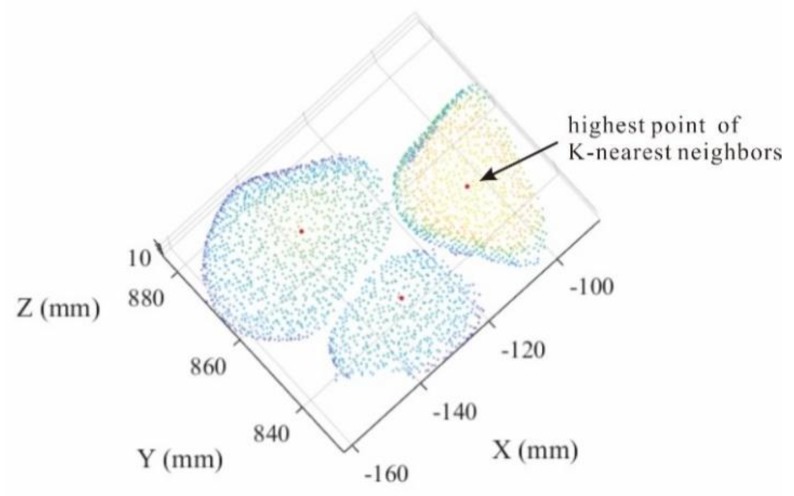
The highest point of K-nearest neighbors.

We first need to run the hill climbing algorithm for regional point set, and find its highest point. In addition, duplicate points that a certain point identified as the highest point by multiple K-nearest neighbors should be combined to prevent the production of redundant points (Figure 6).

**Figure 7:**
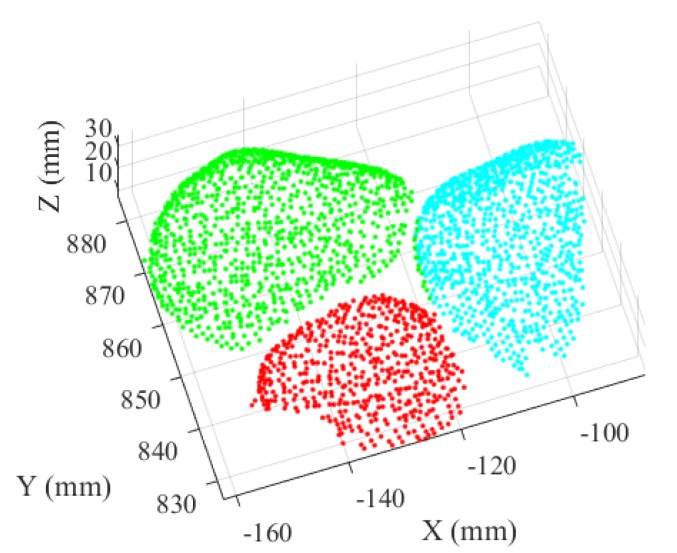
Performance of particle recognition.

Then, the regional area growth is realized through increasing data points based on its radius from the peak to bottom. Meanwhile, the appeared fragmentary regions that identified as small region with different blocks because of its irregular shapes are combined into a similar region and recognized as the point cloud region of this independent block (Figure 7).

## 3. Results and Discussion

### 3.1. Laboratory Physical Model

AS and HC-RG algorithms were used to identify the particle and calculate their radii from point cloud of accumulation surface of three sliding materials (pebbles, gravels, and mixtures).

#### 3.1.1. Pebbles Deposit

**Figure 8:**
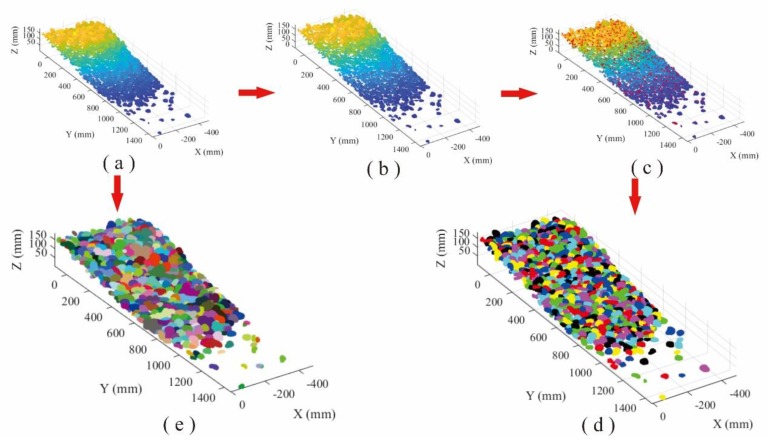
Comparison of recognition performance of pebble loose accumulation: (**a**) point cloud, (**b**) separation processing, (**c**) peak points of each block region, (**d**) recognition using HC-RG algorithm, and (**e**) recognition using AS algorithm.

Totally 843,338 points with XYZ coordinates were generated for the loose accumulation surface of pebbles using portable laser scanner. The AS algorithm was employed to recognize the particles from the accumulation point cloud with 1.09 of alpha value. Data processing lasted 27 s and 648 particles were detected. On the other hand, based on the HC-RG algorithm, the point cloud is firstly separated through moving along the reverse direction of point normal. Then, the point cloud data were clustered into various local regions (637 blocks) based on separation results, and each region represented an individual pebble, and the peak point of the region was found via HC algorithm. The region growing was performed to identify the points belonging to each block region by selecting the peak point as the initial growth seed. The detection results of loose accumulation were obtained, and the total processing time is 15,576 s for HC-RG algorithm (Figure 8d).

**Figure 9:**
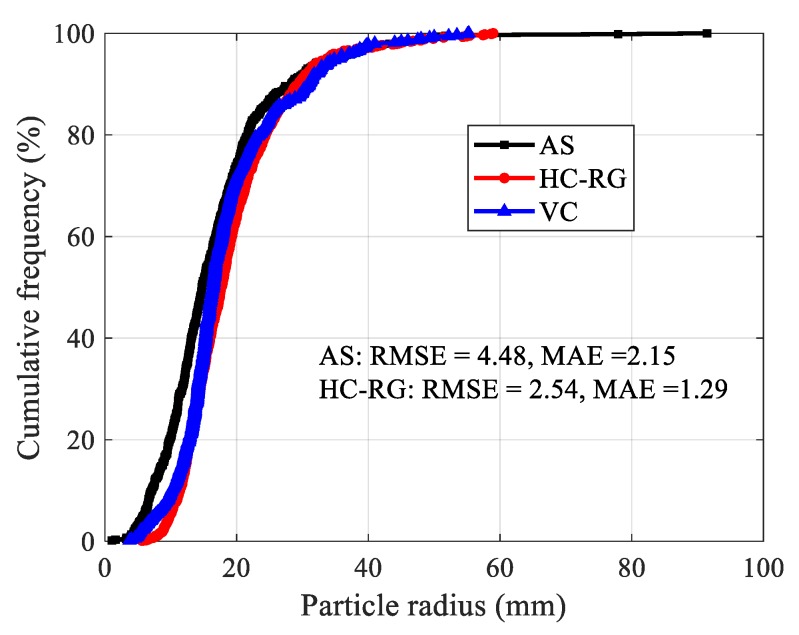
Particle radius distribution of pebbles.

Figure 8d,e illustrates the recognition results from HC-RG and AS algorithms, in which different colors represent different blocks. The processing time for AS algorithm (27 s) is far less than that of HC-RG algorithm (15,576 s). To quantificationally measure the level of confidence or the precision and bias of the obtained results for each method, the root-mean-squared error (RMSE) and the mean absolute error (MAE) are determined according to Equations (6) and (7). Smaller values of RMSE and MAE indicate higher precision.

(6) $$RMSE=\sqrt{\frac{\sum_{i=1}^{n}{\left(p_{pi}-p_{oi}\right)}^{2}}{n}}$$

(7) $$MAE=\frac{1}{n}\sum_{i=1}^{n}\left|p_{pi}-p_{oi}\right|$$

where, *p_pi_* indicates the predicated values of pebbles radius using AS or HC-RG algorithm, *p_oi_* denotes the observed values of pebbles radius measured by vernier caliper (VC), and n is the amount of pebbles. Statistical analysis show that both results are acceptable, and a better agreement is observed between recognition using HC-RG algorithm and real measurement (Figure 9).

#### 3.1.2. Gravels Deposit

**Figure 10:**
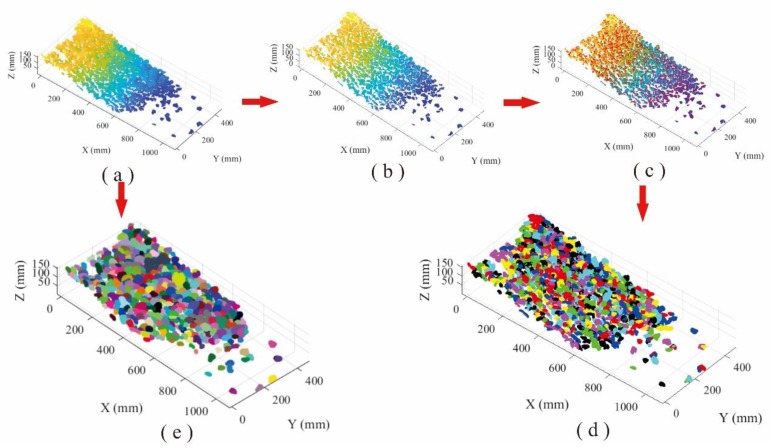
Comparison of recognition performance of gravel loose accumulation: (**a**) point cloud, (**b**) separation processing, (**c**) peak points of each block region, (**d**) recognition using HC-RG algorithm, and (**e**) recognition using AS algorithm.

The point cloud of gravel loose accumulation involves 1,006,987 points. Similarly, AS and Hc-RG algorithms were used to identify the particles and determine their size. Totally, 31,523s and 576 blocks were recognized using HC-RG algorithm, whereas in the AS algorithm, 624 blocks were identified in 40s by specifying alpha value as 1.09 (Figure 10).

**Figure 11:**
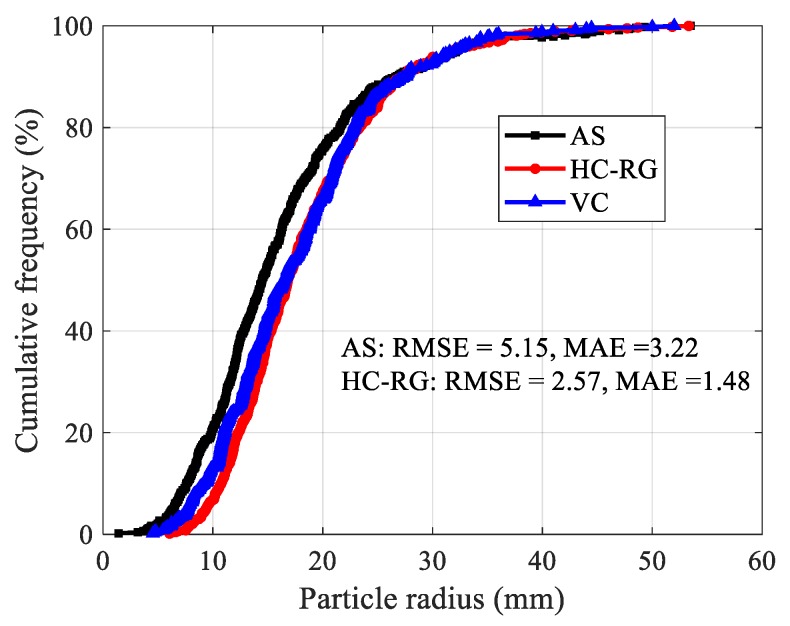
Particle radius distribution of gravel.

Both HC-RG and AS algorithms are capable to accurately identify gravels with different size from the point clouds. However, the result for HC-RG algorithm is consistent with reality better with a lower RMSE and MAE values (Figure 11).

#### 3.1.3. Mixture of Pebbles and Gravels Deposit

**Figure 12:**
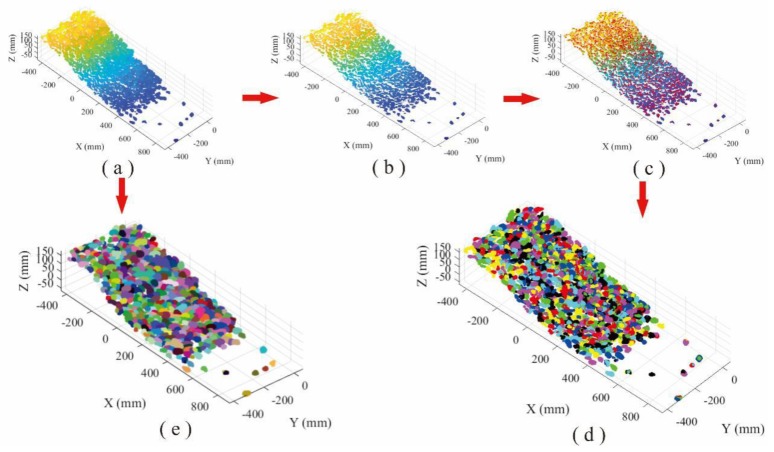
Comparison of recognition performance of mixed material loose accumulation: (**a**) point cloud, (**b**) separation processing, (**c**) peak points of each block region, (**d**) recognition using HC-RG algorithm, and (**e**) recognition using AS algorithm.

**Figure 13:**
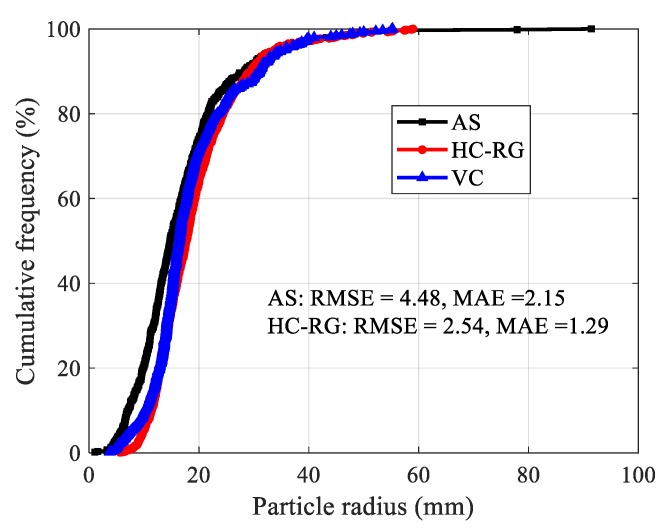
Particle radius distribution of mixed material.

The point cloud of loose accumulation for mixed materials has 1,356,435 points in total. The accumulation was identified using AS algorithm by setting alpha value as 1.09. It cost 27 s for detection, and 870 individual blocks were recognized. Meanwhile, the accumulation was identified by HC-RG algorithm with a 31,523 s processing time, and 588 individual blocks were recognized (Figure 12). A comparison of block size distribution between two methods and real measurement is conducted, and it indicates that results from two algorithms correspond to the manual measurement (Figure 13).

Furthermore, we chose other three parameters to characterize the particle size distribution and test the accuracy of two algorithms, namely, average particle radius (Ra), coefficient of uniformity (Cu), and coefficient of curvature (Cc). Cu and Cc can be determined by,

(8) $$Cu=\frac{D60}{D10}$$

(9) $$Cc=\frac{{\left(D30\right)}^{2}}{D60\times D10}$$

where, *D*10, *D*30, and *D*60 are the particle size that 10%, 30%, and 60% of the particles are finer than those size by volume. The higher the value of the Cu the larger the range of particle sizes. A well graded loose accumulation has a Cc between 1 and 3 [36]. Table 1 shows the comparison of distribution characteristic parameters obtained from different methods between pebbles, gravels and mixture loose accumulation.

From the above-mentioned particle measurement, it is obviously observed that AS algorithm always recognize more number of particles with a smaller size than reality. On the contrary, particle size detected using HC-RG algorithm is slightly larger than real measurement, and has a better agreement with real situation than AS algorithm. The reason can be discussed as follows. (1) For the AS algorithm, a block with irregular shapes and high variance in boundary curvature may be recognized as different blocks, leading to the block number increase and radius reduce. Additionally, there are several limitations when only using a single global alpha value to identify particles from the overall point cloud [37,38]. (2) For the HC-RG algorithm, distinction between adjacent blocks in loose accumulation was enlarged through separation processing which was a useful way to recognize particle accurately. However, there is also a problem existing in the HC-RG algorithm, when some irregular blocks located in a close distance with each other, these particles would be identified as an individual particle. In this manner, small size of particle close to the big one were also considered as a larger group of rock blocks, resulting in larger radius, but fewer number, than real situation.

### 3.2. In-Situ Field Survey in Landslide Region

#### 3.2.1. Upper Part of Landslide Accumulation

**Figure 14:**
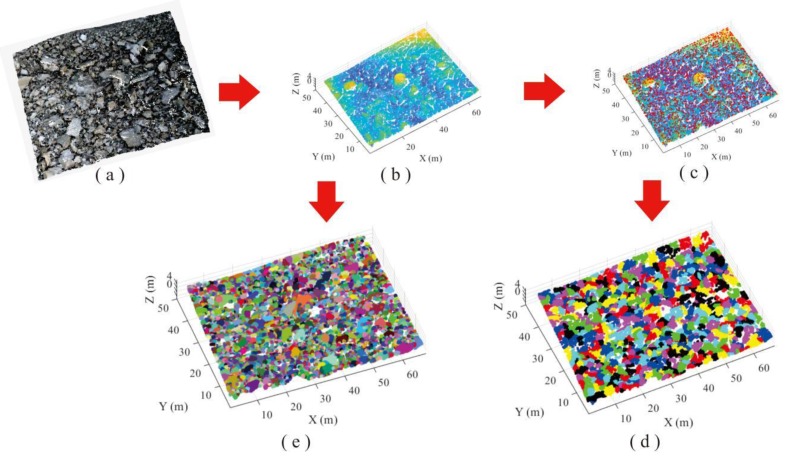
Comparison of recognition performance of upper part of landslide accumulation between two algorithms: (**a**) original point cloud with RGB color, (**b**) point cloud without color, (**c**) separation and peak point searching, (**d**) recognition using HC-RG algorithm, and (**e**) recognition using AS algorithm.

An upper part of the landslide deposit was chosen for block detection using two algorithms, where lots of rock blocks with irregular shapes and various sizes; 292,622 points were generated by TLS for the scanning region. Prior to recognition using AS algorithm, the alpha value was specified as 0.1 m, and 19 s was the cost to identify 5495 blocks. In the HC-RG algorithm, data processing lasted 303 s and 582 blocks were extracted from point cloud (Figure 14).

**Figure 15:**
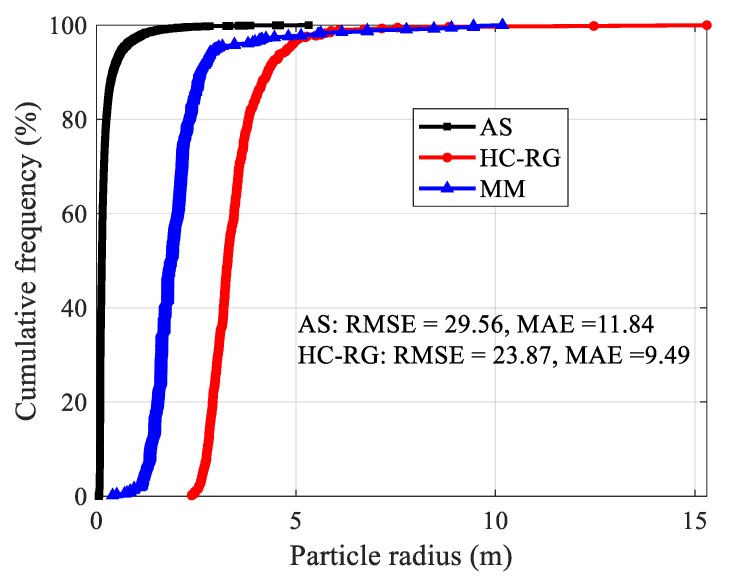
Particle radius distribution of upper accumulation of landslide.

The AS algorithm can identify various blocks, but the identified amount is large, and the radius is small. Compared with AS algorithm, HC-RG algorithm is better in identification, and the results are closer to reality with RMSE = 23.87 and MAE = 9.49. Particularly notable is that it is very difficult and time-consuming to manually measure the blocks size in the landslide deposit. Hereon, the real size was obtained by performing manual measurement (MM) in Geomagic software based on the point clouds (Figure 15).

#### 3.2.2. Lower Part of Landslide Accumulation

**Figure 16:**
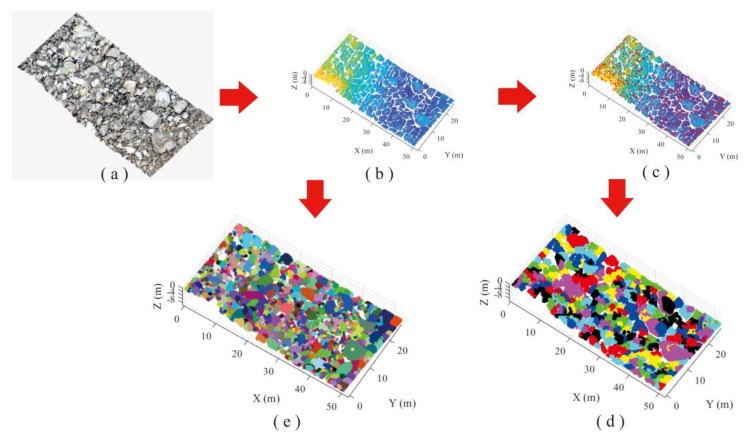
Comparison of recognition performance of lower part of landslide accumulation between two algorithms: (**a**) original point cloud with RGB color, (**b**) point cloud without color, (**c**) separation and peak point searching, (**d**) recognition using HC-RG algorithm, and (**e**) recognition using AS algorithm.

**Figure 17:**
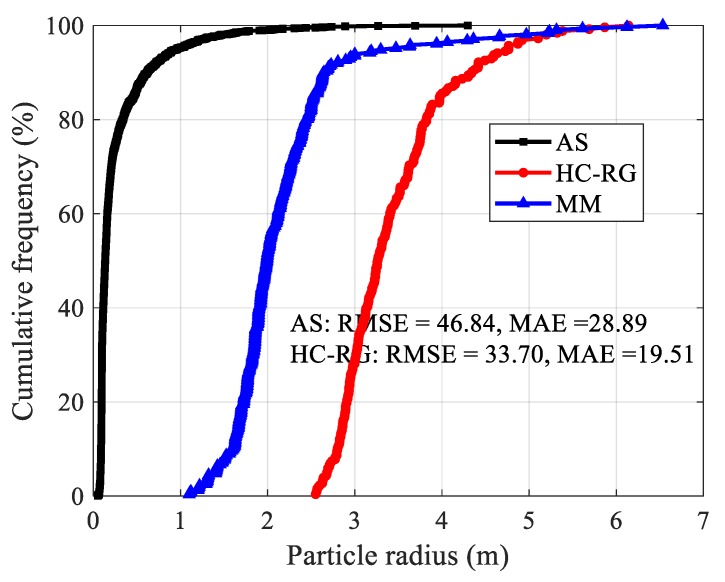
Particle radius distribution of lower accumulation of landslide.

There are 200,306 points totally collected using TLS for the lower part of landslide loose accumulation. Then, it was identified by HC-RG algorithm, the processing time was 230 s and 240 blocks were identified. For AS algorithm, alpha value was set as 0.1. The computational time was 9 s and 1768 blocks were recognized (Figure 16). Similarly, reletively large difference between real measurement and identification results were observed in the comparison, but the HC-RG algorithm had a better recognition than AS algorithm (Figure 17).

Table 2 shows the estimation of three distribution characteristic parameters. On the whole, the average particle size in upper region is larger than that in the lower region, and particles in the upper region have a larger Cu and Cc values than those in lower region. We have to admit that obvious differences of average particle size exist between recognition results and real size. However, the Cu and Cc results obtained from AS and HC-RG algorithms are acceptable. On the other hand, good agreements can be observed in the laboratory physical model due to the controlled conditions.

Two main reasons for analysis errors have been found as follows. (1) More noise data tend to be generated under the field environment when the point clouds are collected using long range TLS, because of the effects from vegetation, humidity, dust in air, microvibration, etc. These non-ideal environmental factors greatly reduce the measuring accuracy and data quality [39,40]. (2) The landslide loose accumulation consists of particles with various size ranges, and there are many small particles formed by extrusion and collision during landslide failure. The gaps among large particles are filled with these small particles, enlarging the recognition difficulty since the particles in accumulation contact tightly with each other [41,42].

## 4. Conclusions

Both AS and HC-RG algorithms used in this study show a high performance, and are capable of identifying the particles from point clouds and determining the amount and radii of particles reasonably, especially under the controlled conditions.

The AS algorithm can accurately identify loose accumulation particles in laboratory conditions by setting a proper alpha value. It takes less time for the identification and characterization process but with lower accuracy than the HC-RG algorithm. By contrast, although the HC-RG algorithm has a lower computing efficiency, it can detect the particles from point cloud with higher level of confidence, and the recognition results match the actual situation better. Nevertheless, acceptable results can be obtained when employing the two algorithms for field survey. However, due to the low quality of point cloud data and complexity of particle geometry and contact status, some errors tend to be produced.

## Figures and Tables

**Table 1:** Comparison of distribution characteristic parameters among pebbles, gravels, and mixtures.

Parameters	Methods	Materials		
		Pebbles	Gravels	Mixture
Ra (mm)	AS	17.06	16.2	16.75
	HC-RG	21.14	18.29	19.31
	MM	18.38	17.59	18.58
Cu	AS	3.19	2.14	2.36
	HC-RG	1.52	1.73	1.7
	MM	2.26	2.05	1.76
Cc	AS	1.15	1.09	1.15
	HC-RG	0.99	0.97	0.96
	MM	1.17	0.99	1.11

**Table 2:** Comparison of distribution characteristic parameters among upper and lower part of landslide deposit.

Parameters	Methods	Location	
		Upper Part	Lower Part
Ra (mm)	AS	242.38	281.96
	HC-RG	3478.13	3430.46
	MM	2162.53	2040.81
Cu	AS	1.72	1.81
	HC-RG	1.24	1.22
	MM	1.51	1.33
Cc	AS	0.76	0.69
	HC-RG	0.97	0.95
	MM	0.96	0.99
